# PLOD3 facilitated T cell activation in the colorectal tumor microenvironment and liver metastasis by the TNF-α/ NF-κB pathway

**DOI:** 10.1186/s12967-023-04809-w

**Published:** 2024-01-06

**Authors:** Min Ding, Cheng Wang, Junhong Hu, Junjun She, Ruoyu Shi, Yixuan Liu, Qi Sun, Haojun Xu, Guoren Zhou, Wenlan Wu, Hongping Xia

**Affiliations:** 1 Department of Pathology & Nanjing Drum Tower Hospital Clinical College & Key Laboratory of Antibody Technique of National Health Commission && Jiangsu Antibody Drug Engineering Research Center, Nanjing Medical University, Nanjing, 211166 China; 2 Zhongda Hospital, School of Medicine, Advanced Institute for Life and Health, Southeast University, Nanjing, 210009 China; 3 Department of General Surgery & High Talent & Center for Gut Microbiome Research, Med-X Institute, The First Affiliated Hospital of Xi’an Jiaotong University, Xi’an, 710061 China; 4 Department of Pathology, Tangdu Hospital, Fourth Military Medical University, Xi’an, 710038 Shaanxi China; 5 Department of Colorectal and Anal Surgery, The First Affiliated Hospital of Zhengzhou University, Zhengzhou, 450000 China; 6 Department of Anatomical Pathology, Singapore General Hospital, Singapore, 169856 Singapore; 7 Department of Pathology, The Affiliated Drum Tower Hospital of Nanjing University Medical School, Nanjing, 210008 Jiangsu China; 8 Jiangsu Cancer Hospital, The Affiliated Cancer Hospital of Nanjing Medical University, Jiangsu Institute of Cancer Research, Nanjing, 210009 China

**Keywords:** PLOD3, Colorectal cancer, Liver metastasis, Proliferation, NF-κB

## Abstract

**Background:**

Colorectal cancer (CRC) has been the third most prevalent cancer worldwide. Liver metastasis is the critical factor for the poor prognosis of CRC. Here, we investigated the expression and role of PLOD3 in CRC.

**Methods:**

Different liver metastasis models were established by injecting PLOD3 stable knockdown or overexpression CT26 or MC38 mouse CRC cells into the spleen of mice to verify the tumorigenicity and metastasis ability in vivo.

**Results:**

We identified PLOD3 is significantly overexpressed in liver metastasis samples of CRC. High expression of PLOD3 was significantly associated with poor survival of CRC patients. The knockdown of PLOD3 exhibited remarkable inhibition of proliferation, migration, and invasion in CRC cells, while the opposite results could be found in different PLOD3-overexpressed CRC cells. Stable knockdown of PLOD3 also significantly inhibited liver metastasis of CRC cells in different xenografts models, while stable overexpression of PLOD3 promotes liver metastasis and tumor progression. Further studies showed that PLOD3 facilitated the T cell activation in the tumor microenvironment and affected the TNF-α/ NF-κB pathway.

**Conclusions:**

This study revealed the essential biological functions of PLOD3 in colon cancer progression and metastasis, suggesting that PLOD3 is a promising translational medicine target and bioengineering targeting PLOD3 overcomes CRC liver metastasis.

**Graphical Abstract:**

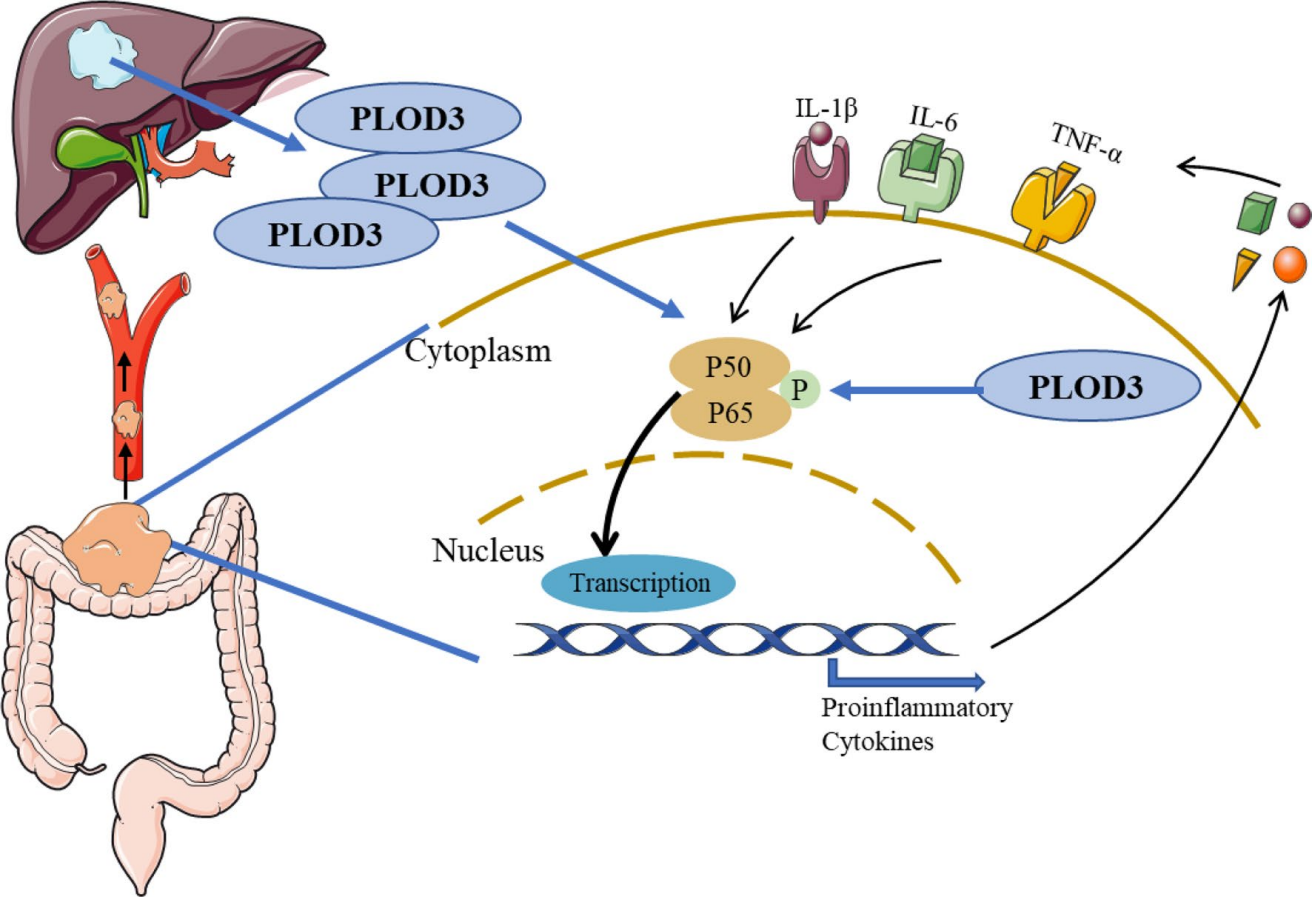

**Supplementary Information:**

The online version contains supplementary material available at 10.1186/s12967-023-04809-w.

## Introduction

Colorectal cancer (CRC) has been the third most prevalent worldwide after breast and lung cancer. In 2020, there were 19.29 million new cancer cases and 9.96 million deaths worldwide, including 4.569 million new malignancies and 3.003 million deaths in China [1]. Distant metastases occurred in 90% of patients who died of CRC. Once metastasis occurs, the 5-year survival rate drops to 13.1%. The early diagnosis and successful control of metastatic initiation and growth are crucial for assessing metastatic potential and the effectiveness of cancer prevention and treatment for colon cancer. Numerous epidemiologic studies have demonstrated the association between cancer occurrence and various lifestyle factors, such as adiposity, physical activity, and diet, resulting in an increased risk of colorectal cancer incidence [2–4]. Though early surgical resection could achieve the maximal therapeutic result, many people suffer from distant metastases and their survival is not ideal yet [5]. Therefore, it is critical to explore the molecular mechanisms of CRC tumorigenesis and liver metastasis, reducing mortality and enhancing the quality of life.

Collagen is a crucial component of the extracellular matrix [6]. Procollagen-lysine, 2-oxoglutarate 5-dioxygenases (PLODs) family members participate in lysine hydroxylation to achieve collagen deposition and cross-linking [7]. PLODs have been reported as three isoforms (PLOD1-3) [8]. PLOD3 can be defined as a multifunctional enzyme with collagen-galactosyltransferase, lysine hydroxylase, and glucosyltransferase. Mutations and overexpression of PLODs can cause the development of various malignancies and promote tumor metastasis. Here, we identified PLOD3 is significantly overexpressed in liver metastasis samples of CRC by the gene expression profile of clinical primary CRC and liver metastasis tissue samples. Numerous studies have shown that PLOD3 develops various tumors [9]. Abnormal expression of PLOD3 is related to unfavorable prognosis in pulmonary carcinoma [10, 11], hepatocellular carcinoma, gastric cancer [8, 12], renal cell carcinoma [13], glioma [14] and connective tissue disease [15]. Nonetheless, the expression pattern and function of PLOD3 in colorectal cancer were still uncertain. How PLOD3 participates in the progression and liver metastasis of CRC has not been well investigated. Herein, this study illustrated the biological function and mechanism of the PLOD3 gene to regulate the process of liver metastasis of colorectal cancer.

NF-κB is associated with many pathological processes and is a transcription factor associated with inflammation. The NF-κB pathway is divided into classical and non-classical activation. The classical pathway is that various signals activate NF-κB by degrading IκBs, and the activated NF-κB then enters the nucleus to bind to DNA [16]; the non-classical pathway, on the other hand, is achieved through the proteolytic processing of p100. When the NF-κB classical pathway is activated, it can protect the host from invading anti-pathogenic microorganisms, promote cell proliferation and inhibit cell apoptosis by secreting cytokines (TNF-α, IL-1β and IL-6, etc.). It can increase the expression of pro-angiogenic genes such as VEGF (vascular endothelial growth factor), MCP-1 (Monocyte chemotactic protein-1), vascular cell adhesion molecules (VCAM) and stromal Matrix metalloproteinases (MMP) expression to promote tumor cell migration, to achieve tumorigenic development [17]. Many inflammatory factors, oncogenic and pro-oncogenic agents, and tumor microenvironment can activate NF-κB. NF-κB and its regulated proteins are associated with tumorigenesis, proliferation, anti-apoptosis, invasion, angiogenesis and metastasis [18–24].

The current study aimed to identify the underlying molecular mechanisms of PLOD3-influenced CRC proliferation and migration/invasion in vitro. We further investigated the critical role of PLOD3 expression on the liver metastasis of CRC using different liver metastasis mice models of CRC in vivo. Our study also clarified the potential link between PLOD3 and the TNFα/NF-κB signaling pathway in the tumor microenvironment. This study revealed the essential biological functions of PLOD3 in colon cancer progression and metastasis, suggesting that PLOD3 is a promising therapeutic target for colorectal cancer liver metastasis.

## Materials and methods

Additional methods are described in the Supporting Materials and Methods online.

### Animal models

All the animals were approved by the Animal Core Facility of Nanjing Medical University and performed according to the institutional guidelines. To establish animal models of colorectal cancer with liver metastasis, the colon cancer cells (2 × 10^5^, suspended in 100 μl of PBS) containing PLOD3 knockdown plasmid or control plasmid were injected into the spleen by using an insulin syringe and compressed the wound for three minutes to stop the bleeding. Fluorescence was monitored 2 weeks away from injection using the IVIS Spectral In vivo Imaging System (IVIS Lumina System, PerkinElmer USA). After 4 weeks, the mice were euthanized and sacrificed. Meanwhile, peripheral blood, liver and tumor samples were collected for further analysis.

### Lentivirus transfection and construction of stable cell lines

To silence PLOD3, a PLOD3 knockdown stable cell line was constructed with pLKO.1-shScramble, pLKO.1-shPLOD3 vector (purchased from Sigma-Aldrich). Briefly, lentiviral packaging was performed by co-transfection of shRNA plasmids and packaging plasmids (pMD2.5G and psPAX2) into HEK293T cells. Viral supernatants were collected 48 h after transfection. Cells were plated in 6-well plates and incubated with a virus-containing medium for 48 h. Cell selection was then performed with a medium containing 2 μg/ml puromycin. PLOD3 knockdown efficiency was confirmed by immunoblotting and qPCR after 7–14 days.

The shRNAs targeting PLOD3 sequences were as follows: 5ʹ-CCGGGAGGATATGATCATCAT-3ʹ(shPLOD3-1); and 5ʹ-TCCGCGTGCCTGAACTGAATA-3ʹ (shPLOD3-2).

To construct PLOD3 overexpression cell lines. PLOD3 was amplified by PCR and ligated into the lentiviral overexpression vector pLenti-CMV-GFP/puro. Lentiviral vectors were packaged in HEK293T using transfection reagents along with pMD2.G and psPAX2. Lentivirus was harvested 48 h after co-transfection and used to infect cells. Infected cells were screened in a medium containing puromycin (2 μg/ml) for 7–14 days. Stable transfectants were selected and confirmed by western blot analysis.

### Statistical analysis

Statistical analysis was performed using GraphPad Prism 8. All experiments were repeated more than three times, and the results were expressed as mean ± SD. Differences in PLOD3 expression between cancerous and paracancerous tissues were analyzed by paired tests. Comparisons between the two groups were analyzed by Student’s t-test (two-tailed). The Chi-square test was applied to evaluate the relationship between PLOD3 expression and clinical characteristics. P < 0.05 was regarded as statistically different.

## Results

### PLOD3 is identified as having high expression in colon cancer with liver metastasis and is associated with poor survival

**Fig. 1:**
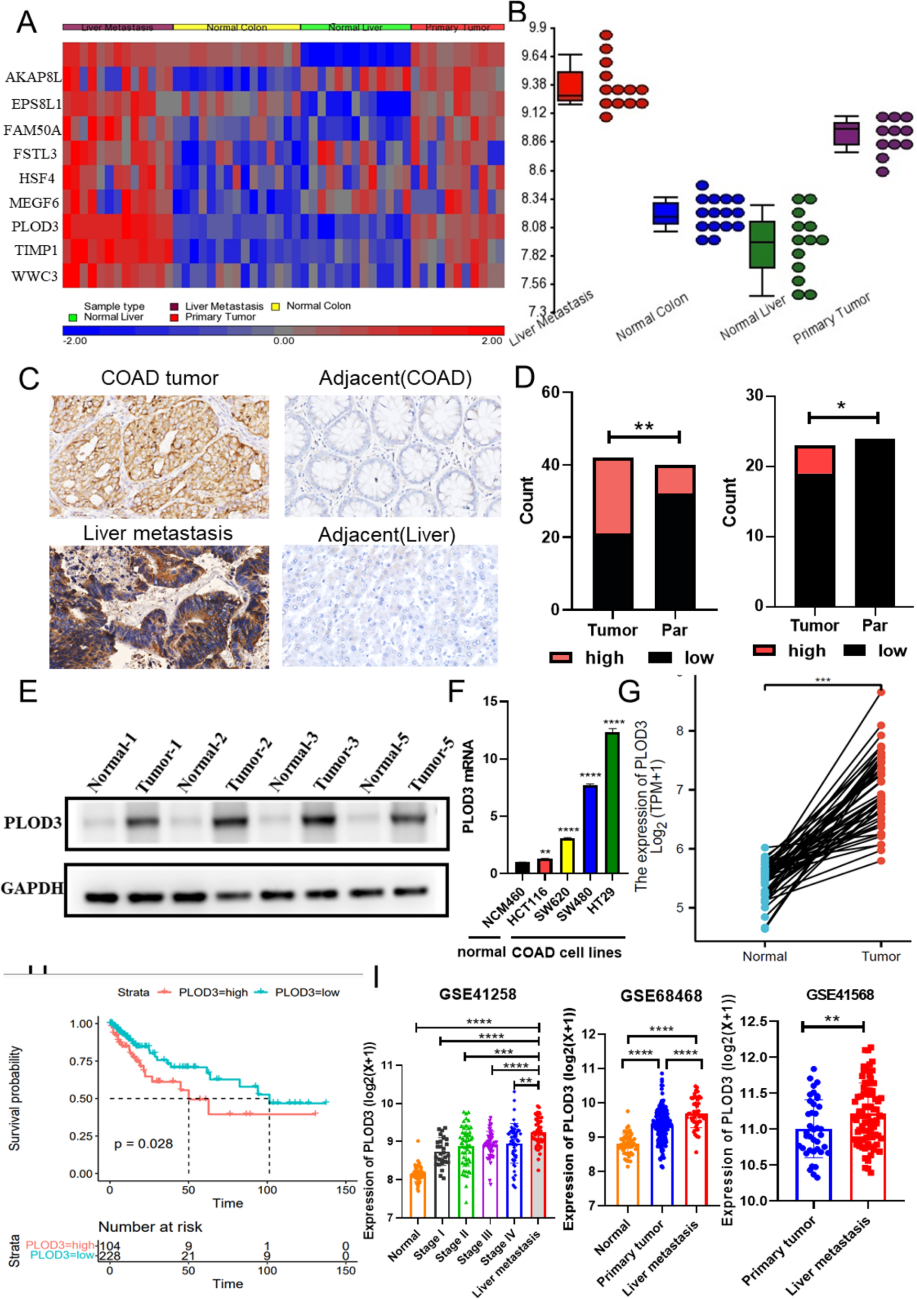
PLOD3 is highly expressed in colon cancer liver metastasis and is correlated with low survival rates. **A** The heatmap showed that we identified a panel of significantly different genes in liver metastasis tissues and primary CRCs by RNA sequencing. **B** The expression difference of PLOD3 was further validated in tissue samples by RT-qPCR analysis. **C**, **D** The expression and quantification of PLOD3 were further validated in CRC and para-carcinoma tissue samples by immunohistochemical staining assay. Scale bars, 20 μm (400 ×), were shown in the right corner of each picture. Graphical illustration of statistical PLOD3 distribution in CRC and liver metastasis patients. **E**, **F** The western blot and RT-qPCR analysis further confirmed that CRC tumor tissues and cell lines. **G** PLOD3 was also highly expressed in the tumor compared to adjacent tissue in the TCGA dataset. **H** High PLOD3 expression was associated with low survival of patients. I Comprehensive analysis of PLOD3 expression in public microarray profiling datasets from the Gene Expression Omnibus (GEO) database (GES41258, Mann–Whitney test; GES68468 paired t-test; GSE41568 paired t-test). Data are shown as the mean ± SEM (three independent experiments). *P < 0.05; **P < 0.01; ***P < 0.001)

We have sequenced a panel of section tissue samples from CRC with liver metastasis. By bioinformatics analysis, we identified a panel of genes that are significantly different in liver metastasis tissues and primary CRCs (Fig. 1A). Among them, the expression difference of PLOD3 was further validated in tissue samples by RT-qPCR analysis (Fig. 1B) and immunohistochemistry (IHC) staining assay (Fig. 1C and D). Figure 1D is the result of our statistical analysis of Fig. 1C, which counts the expression levels of PLOD3 in tumors, adjacent tissues, and liver metastases. The western blot and RT-qPCR analysis further confirmed that CRC tumor tissues and cell lines show a higher PLOD3 expression than the matched normal tissues or colon cell line (Fig. 1E and F). We also analyzed 41 cancerous and adjacent tissues derived from The Cancer Genome Atlas (TCGA) cohort. As the data has shown in Fig. 1G, PLOD3 was also highly expressed in the tumor compared to adjacent tissue. High PLOD3 expression was associated with low survival (Fig. 1H). We also comprehensively analyzed PLOD3 expression in public microarray profiling datasets from the Gene Expression Omnibus (GEO) database (GSE41258, GSE 68468, GSE41568) (Fig. 1I). Furthermore, the same result can also be observed from Additional file 1: Table S1 that the high expression of PLOD3 was correlated with clinical stage and M type, but not with other clinical parameters such as age, sex, and N-type T-type. In conclusion, these data reveal that PLOD3 expression is upregulated in CRC and liver metastasis, suggesting that PLOD3 may be a liver metastasis promoter in CRC.

### PLOD3 strengthens the CRC cell growth and proliferation in vitro

**Fig. 2:**
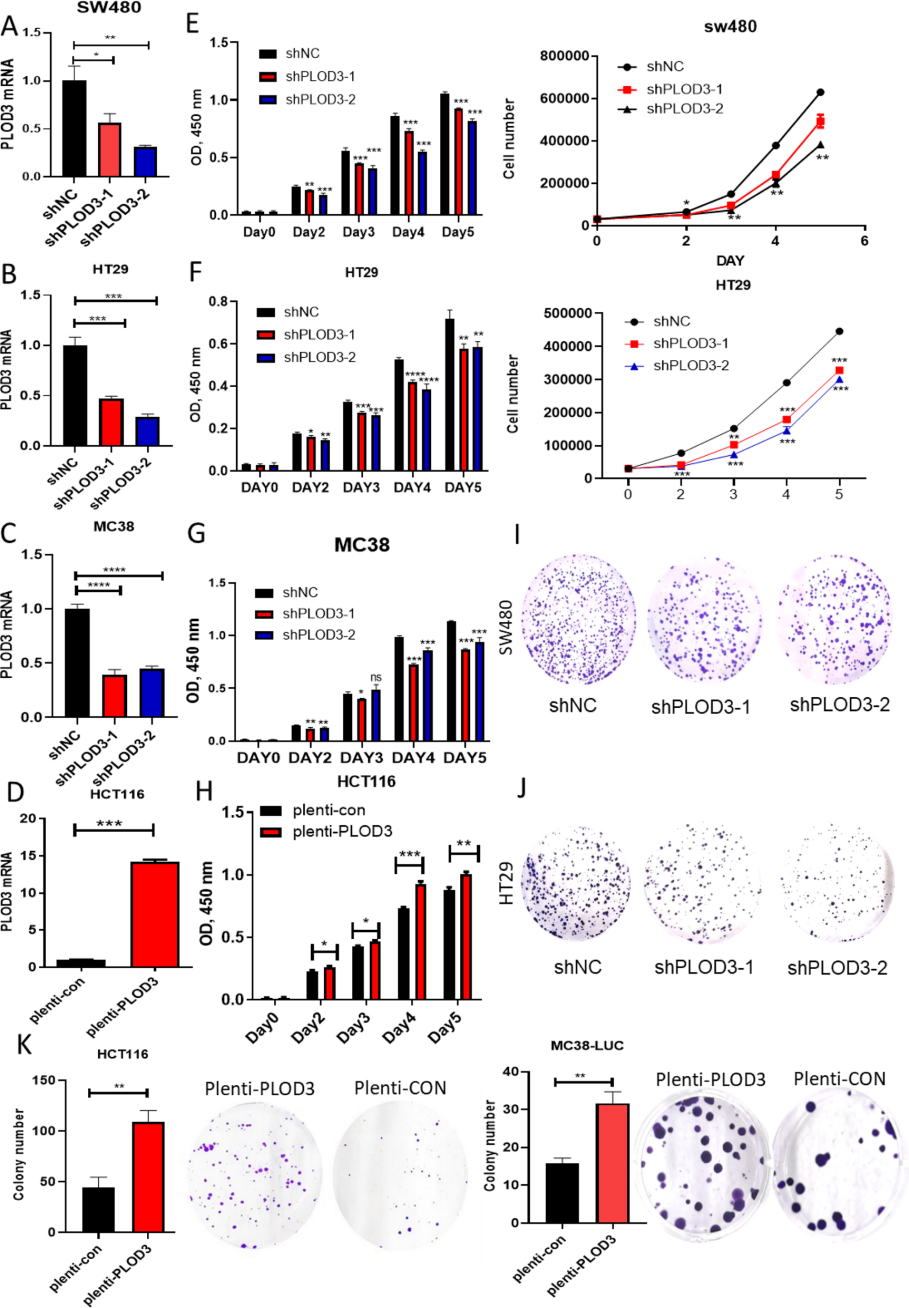
PLOD3 strengthens the CRC cell proliferation in vitro. **A**, **B**, **C** qRT-PCR analysis of PLOD3 mRNA levels normalized to GAPDH in the CRC cell lines stably transduced with PLOD3-targeting shRNA or control shRNA; **D** mRNA levels of PLOD3 upon PLOD3 overexpression by lentivirus‐PLOD3; **E**, **F**, **G**, **H** CCK-8 assays to determine the proliferation of PLOD3 knockdown or over-expression in the CRC cells; I–K Colony-forming assays to determine the effects of PLOD3 overexpression or knockdown on the growth of CRC cells. Data are shown as the mean ± SEM (three independent experiments). *P < 0.05; **P < 0.01; ***P < 0.001)

To explore the role of PLOD3 in the cell growth and proliferation promotion of CRC cells, we established overexpressing CRC cell lines stably expressing PLOD3 using the pLenti-CMV-PLOD3 plasmid. In contrast, we constructed the PLOD3 knockdown cells via a lentiviral vector of shRNA-PLOD3. The knockdown efficiency of PLOD3 in SW480, MC38 and HT29 was verified by western blot and qPCR (Fig. 2A–D). First, we used CCK8 to detect the proliferative activity of CRC cells after PLOD3 knockdown, and the results showed (Fig. 2E–G) that knockdown of PLOD3 could inhibit CRC proliferation. Then, we observed increased proliferative activity in HCT116 cells overexpressing PLOD3 compared to control cells (Fig. 2H). In addition, colony formation assays further confirmed that knockdown of PLOD3 inhibited colony formation in CRC cells (Fig. 2I, J, K). In conclusion, PLOD3 enhances the proliferative capacity of CRC cells in vitro.

### PLOD3 promotes CRC cell migration and invasion in vitro

**Fig. 3:**
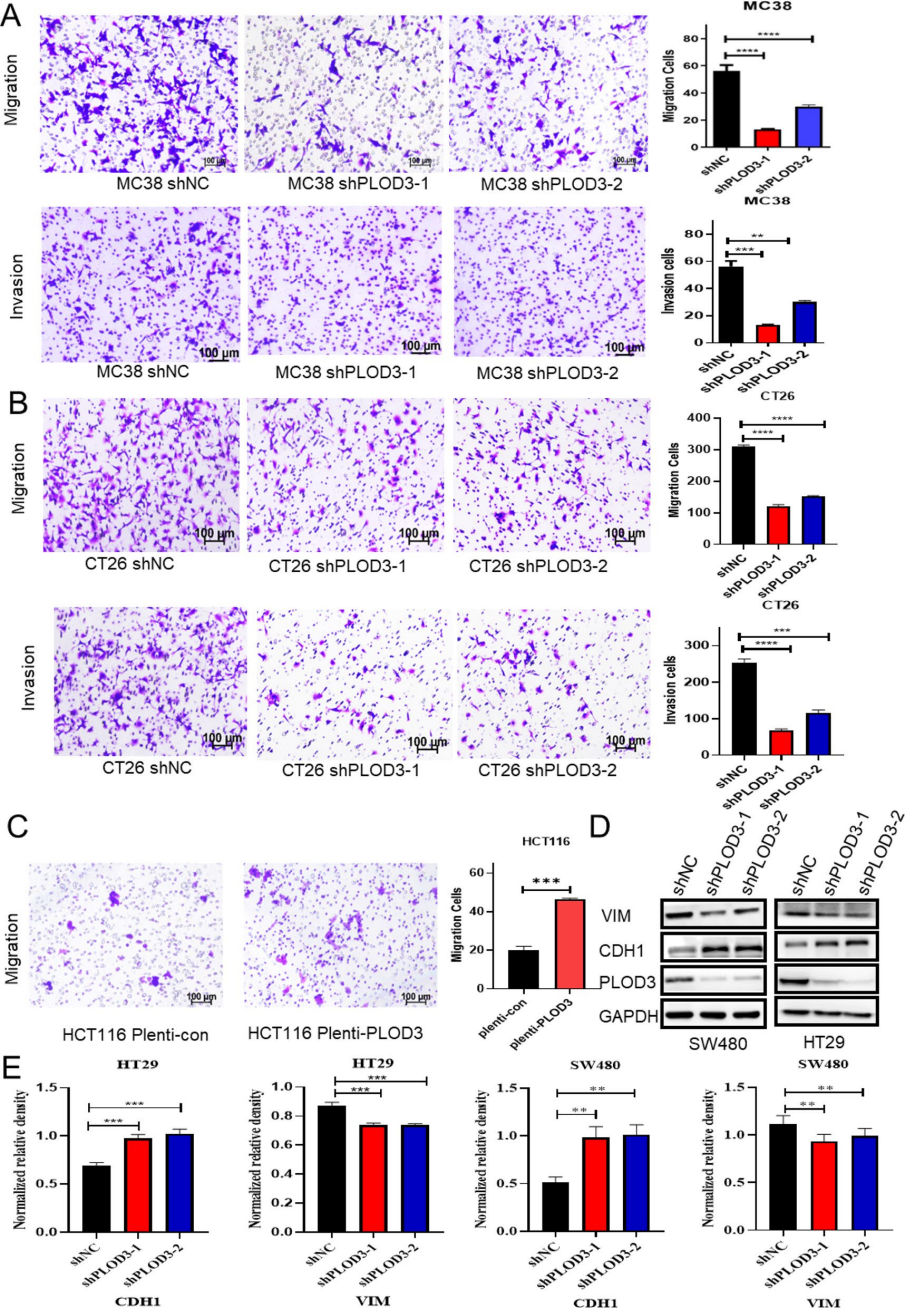
PLOD3 promotes CRC cell migration and invasion. **A**–**C** Transwell filter migration and invasion assays to determine the PLOD3 knockdown or overexpression on the migration ability of CRC cells; **D** Western blotting to measure the expression of VIM and CDH1 after PLOD1 knockdown. **E** The quantification and statistical analysis on the expression levels of CDH1 and VIM and displayed them in the form of histograms. Data are shown as the mean ± SEM (three independent experiments). *P < 0.05; **P < 0.01; ***P < 0.001)

The clinical data indicate that high expression of PLOD3 is involved in distant metastasis. So, we next investigated the effect of PLOD3 on the migration and invasion of CRC cells by transwell assay. The results showed that the migration and invasion ability of MC38 (Fig. 3A) and CT26 (Fig. 3B) cells were significantly decreased after PLOD3 knockdown, while PLOD3 overexpression greatly increased the migration and invasion ability of HCT116 (Fig. 3C) cells. Epithelial-mesenchymal transition (EMT) is essential for the migration and invasion of cancer cells [25–27]. Therefore, to elucidate the potential mechanism by which PLOD3 induces cell migration and invasion, we further investigated the effect of PLOD3 on EMT-related proteins by western blot and showed that PLOD3 knockdown suppressed migration and invasion of CRC cells by attenuating EMT progression, including downregulation of VIM and upregulation of CDH1 (Fig. 3D). We performed quantification and statistical analysis on the expression levels of CDH1 and VIM and displayed them in the form of histograms (Fig. 3E). Overall, PLOD3 facilitated the migration and invasion of CRC cells.

### PLOD3 deficiency induces G2/M cell cycle arrest but does not affect apoptosis

**Fig. 4:**
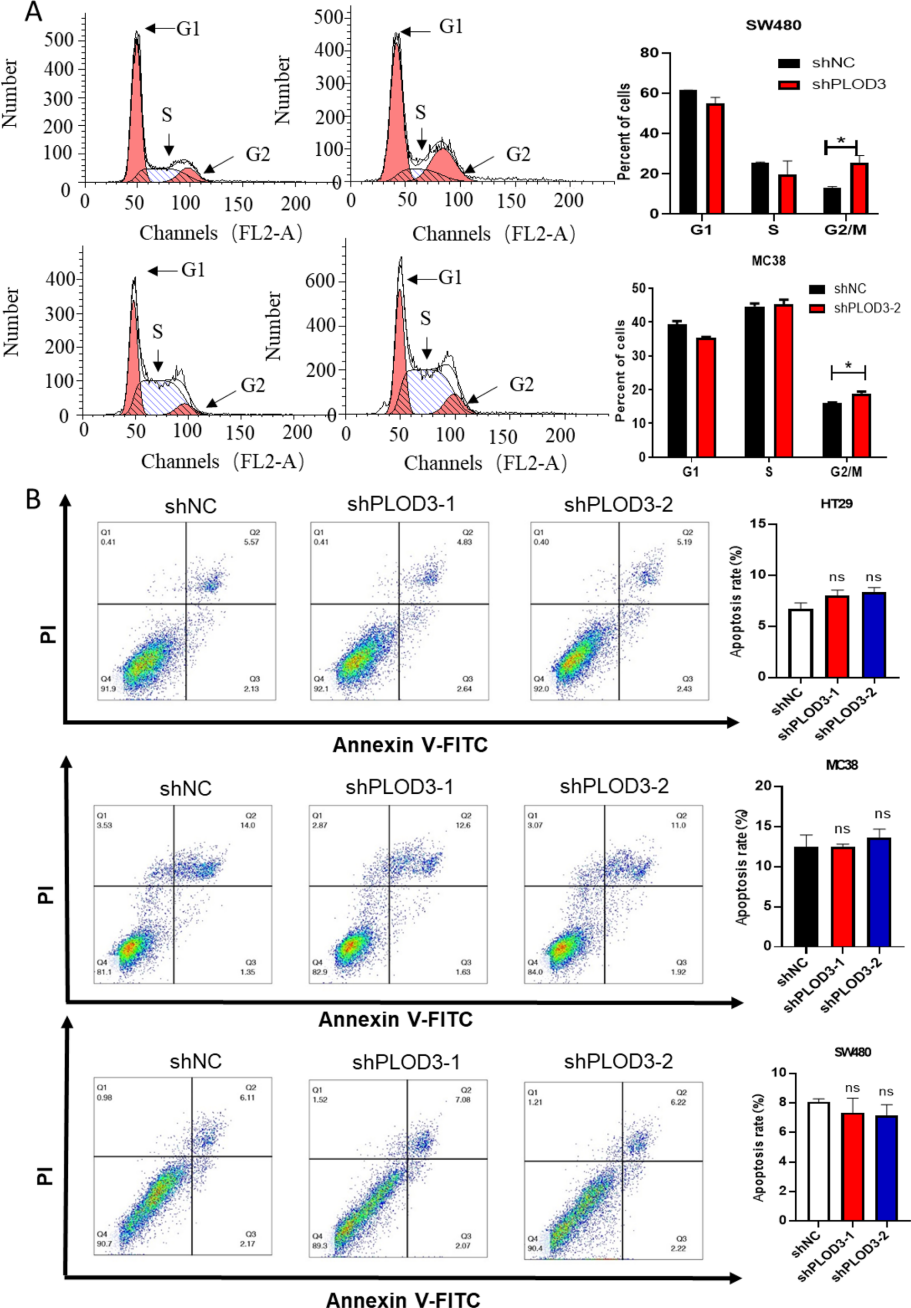
PLOD3 deficiency induces G2/M cell cycle block but not affect apoptosis. **A** The cell cycle distribution in each test group was analyzed by flow cytometry, **B** Propidium iodide staining and flow cytometry. Results show mean ± SD of three independent experiments. Data are shown as the mean ± SEM (three independent experiments). *P < 0.05; **P < 0.01; ***P < 0.001)

The cell cycle progression and apoptosis regulate cell proliferation. We performed flow cytometry assays to assess the effect of PLOD3 on the CRC cell cycle and apoptosis [28]. We observed a significant improvement in the number of cells in the G2/M phase in the SW480 and MC38 cells with the PLOD3 knockdown group compared to the respective control cells (Fig. 4A). We also examined the number of apoptotic cells in both groups using flow cytometry analysis, which showed no significant change in the number of PLOD3 knockdown groups (Fig. 4B). Therefore, these data suggest that PLOD3 knockdown may affect the cell cycle through G2/M blockade.

### PLOD3 exaggerates the CRC liver metastasis in vivo

**Fig. 5:**
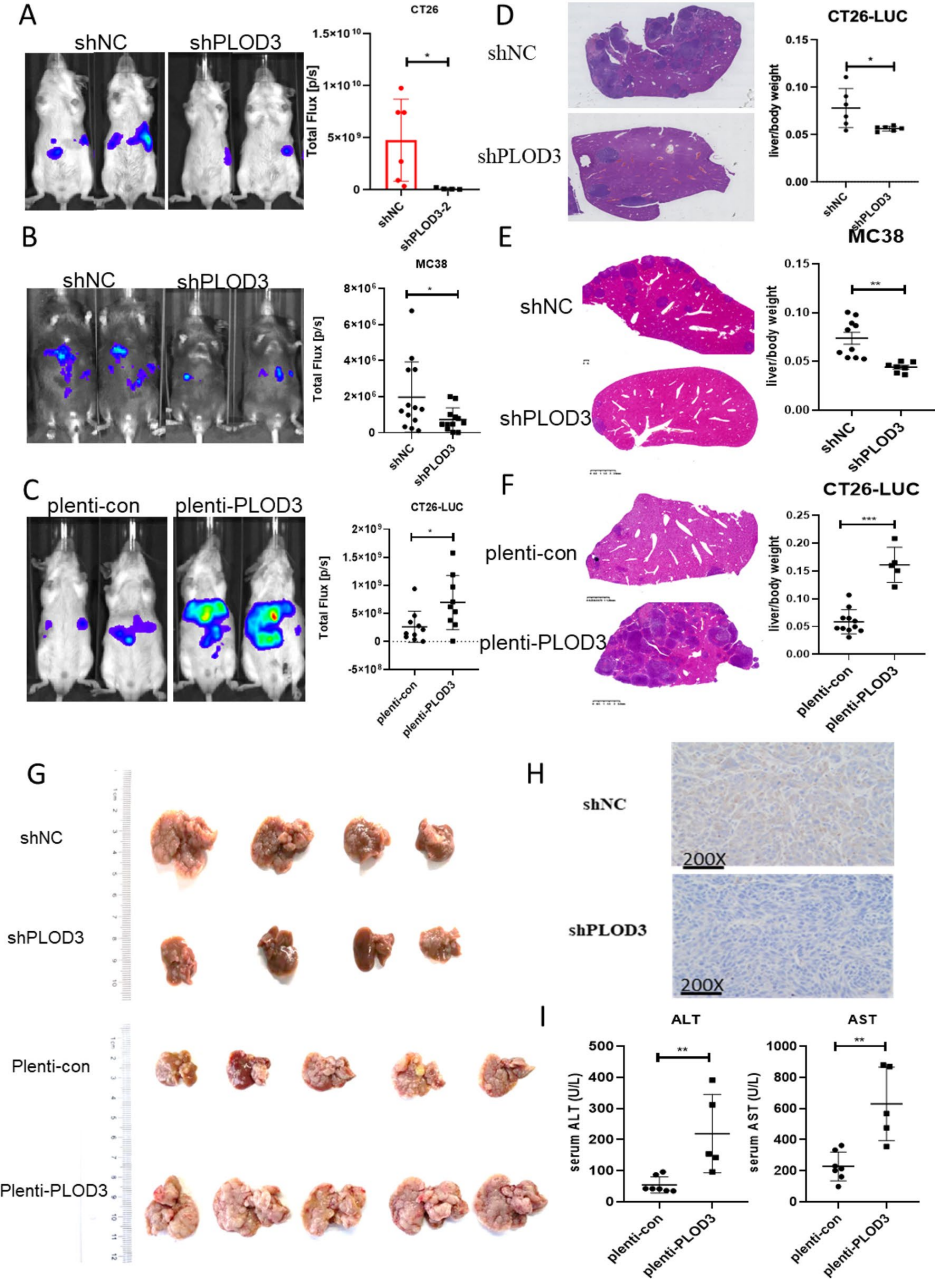
PLOD3 exaggerates the CRC liver metastasis. **A**–**C** Bioluminescence on days 14 post-CT26/MC38-luciferase cell injection. Quantification of the photon flux ratio per mouse at each time point (n ≥ 5 mice per group); **D**, **E**, **F** Hepatic H&E staining of mouse liver with liver metastases. Weights of liver and whole body in shNC group and shPLOD3 group were measured and the results are expressed as the liver weight to body weight ratio; **G** Imaging of metastatic nodules in liver specimens from metastatic models established by spleen injection of CT26 cells. **H** IHC to detect the expression of PLOD3 in mouse liver with liver metastases; **I** Concentration of the mouse serum liver function markers ALT and AST on day 14 after CT26 injection (n ≥ 5 mice per group); Data are shown as the mean ± SEM (three independent experiments). *P < 0.05; **P < 0.01; ***P < 0.001)

The liver is the most common target for colorectal cancer metastasis. To identify the effect of PLOD3 in CRC liver metastasis, we established the MC38 and CT26 cell liver metastasis models. MC38 and CT26 knockdown/overexpression cell lines were constructed by lentivirus, and then these cells were injected into the spleen of mice to enable the efficient spread of cancer cells to the liver. Metastatic lesions in the liver were monitored using in vivo bioluminescence on day 14 after tumor cells injection. The increase in bioluminescence intensity was significantly suppressed in the livers of the shPLOD3 group compared to the livers of shNC group. It was significantly enhanced in the PLOD3 overexpression group (Fig. 5A, B, C).

After the mice were euthanized, the liver/body weight ratio was measured, and the results showed that compared with the control group, the liver weight of the mice in the PLOD3 overexpression group was significantly increased (Fig. 5F). The entire liver had diffuse tumor foci. In contrast, shPLOD3 mouse livers showed fewer tumor foci (Fig. 5G). Moreover, the immunohistochemical staining result suggested that PLOD3 expression was down-regulated in the shPLOD3 group (Fig. 5H). The results of tissue H&E staining showed that the biological structure of the liver of the mice in the shNC group was destroyed and there were more metastatic lesions, while the liver of the shPLOD3 mice maintained normal structure and significantly reduced the number of metastatic lesions. When we overexpressed PLOD3, the results were reversed (Fig. 5D). We analyzed several markers, such as ALT and AST in serum, to evaluate the liver function damage. We found a significant increase in ALT and AST in the PLOD3 overexpression group compared to the control group (Fig. 5I). Overall, this evidence suggests that the knockdown of PLOD3 dramatically decreases liver cancer colonies in vivo and preserves liver function.

### PLOD3 facilitated the T cell activation in the tumor microenvironment

**Fig. 6:**
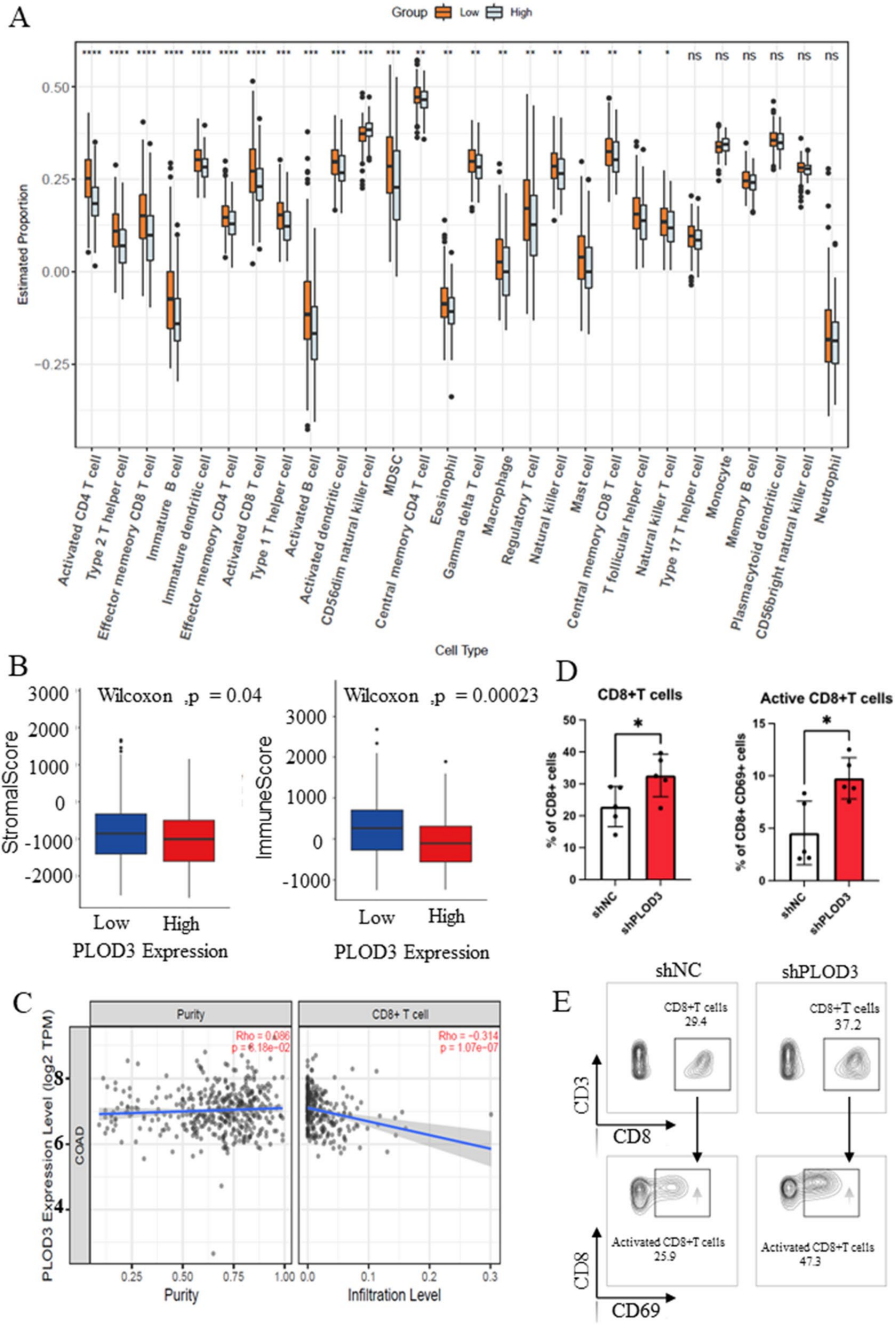
PLOD3 facilitated the T cell activation in the tumor microenvironment. **A** The enrichment score of the 28 kinds of immune cells was evaluated in the high PLOD3 expression and low PLOD3 expression using ssGSEA analysis. **B** ESTIMATE assessed stromal and immune scores in the high PLOD3 expression and low PLOD3 expression groups. **C** The relationship between the mRNA expression of PLOD3 and immune cells was obtained from the Tumor Immune Estimation Resource (TIMER2.0, http://timer.cistrome.org/). **D** Flow cytometric enumeration of CD8 T cells in mouse peripheral blood of the shNC group and shPLOD3 group (n ≥ 5 mice per group). **E** Flow cytometry of CD8 T cells in mouse tumor tissue of the PLOD3-knockdown and the control. Data are shown as the mean ± SEM (three independent experiments). *P < 0.05; **P < 0.01; ***P < 0.001)

To investigate the role of PLOD3 in the CRC tumor microenvironment (TME). We used ssGSEA (single-sample gene set enrichment analysis) to assess each sample’s enrichment fraction of 28 immune cell subsets infiltrated. We found that the infiltration levels of activated CD4 T cells, type 2 helper T cells, effector memory CD8 T cells, activated CD8 T cells, type 1 helper T cells, activated B cells, activated dendritic cells and γδ T cells were significantly higher in the PLOD3 low expression group than in the PLOD3 high expression group(Fig. 6A). Therefore, most immune cells' infiltration in tumor immune activation is elevated in the low-expression PLOD3 group.

Next, we assessed the stromal score and immune score in both groups using ESTIMATE, and the results showed a higher relative content of stromal or immune cells in the TME of the PLOD3 low expression group (Fig. 6B). Next, we used TIMER2.0 to explore the relationship between immune cell infiltration and PLOD3 expression, and the results showed that PLOD3 mRNA levels in COAD were negatively correlated with infiltration of CD8+ T cells (r = − 0.314) and CD4+ T cells (r = − 0.178) (Fig. 6C).

To further understand the effects of PLOD3 on CRC liver metastasis, we detected the activation status of peripheral blood CD8+ T cells in mice with colon cancer liver metastases by flow cytometry. We showed that CD8+ T cells and activated CD8 T cells were significantly increased in the shPLOD3 group (Fig. 6D). Similarly, we also examined the activation status of CD8+ T cells in mouse tumor tissues and showed a significant increase in CD8+ T cells and activated CD8 T cells in the shPLOD3 group (Fig. 6E). These results suggest that knockdown of PLOD3 improves the tumor microenvironment of colon cancer liver metastases.

### PLOD3 accelerated the progression of CRC via TNFα/NF-κB signaling pathway

**Fig. 7:**
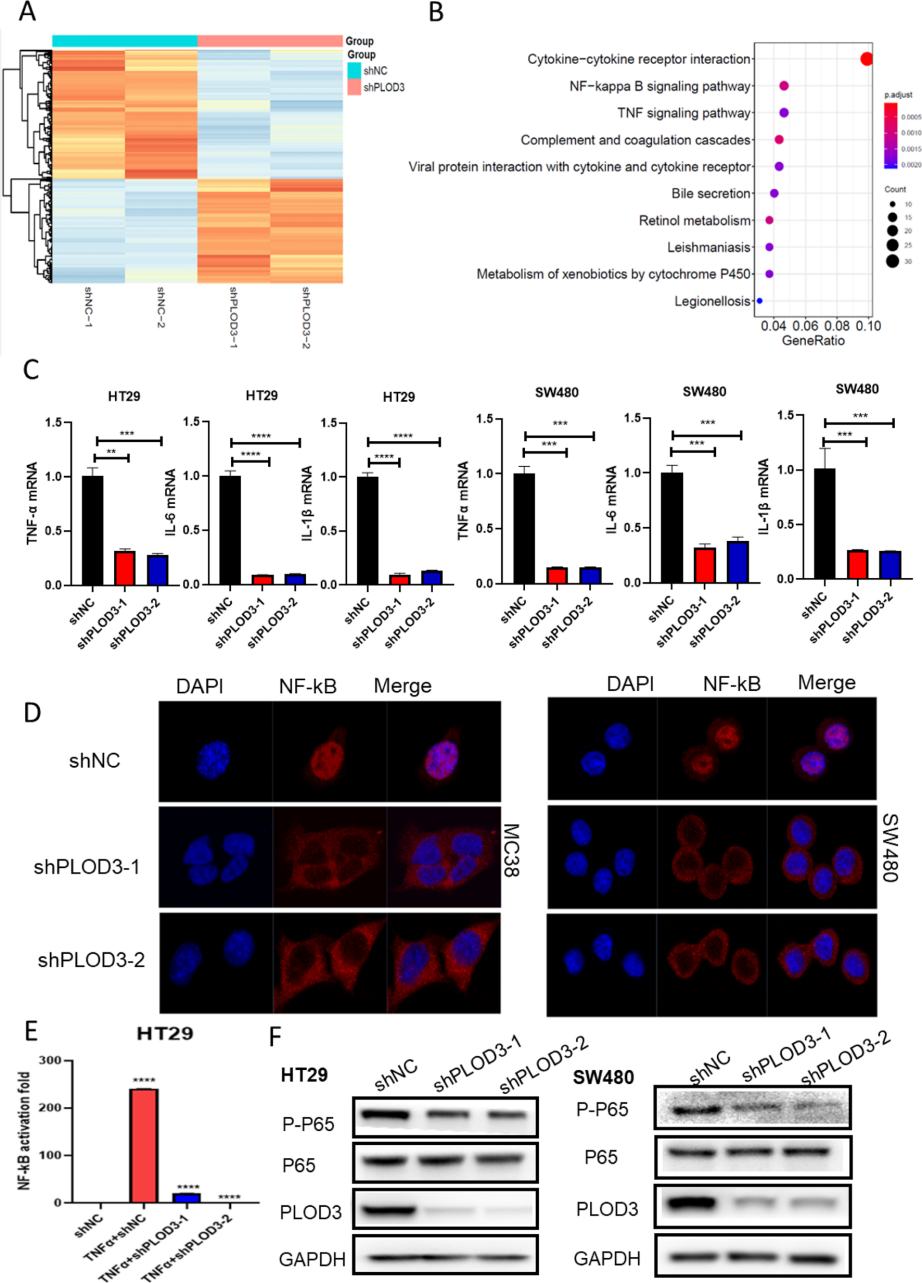
PLOD3 accelerated the progression of CRC via TNFα/NF-κB signaling pathway. **A** Heatmap representation of genes differentially expressed in PLOD3-knockdown HT29 cells identified by RNA-seq. **B** GO analysis of the genes that were uniquely downregulated. **C** qRT-PCR analysis of TNF-a, IL-6 and IL-1b mRNA levels normalized to GAPDH in the CRC cell lines stably transduced with PLOD3-targeting shRNA or control. **D** Immunofluorescence staining of NF-κB (Red) and DAPI (Blue) in MC38 and SW480 cells analyzed by Single Photon Laser Scanning Confocal Microscopy. Magnification, × 160. **E** Luciferase reporter assay indicated that the knockdown of PLOD3 suppressed the p-NF-κB luciferase activity in HT29 cells. (F) Western blot to measure the protein expression of NF-κB pathway after PLOD3 knockdown. Data are shown as the mean ± SEM (three independent experiments). *P < 0.05; **P < 0.01; ***P < 0.001

To explore the molecular mechanism of PLOD3 in CRC, we used RNAseq to analyze and compare the differentially expressed genes between control and PLOD3 knockdown groups in HT29. Genes with more than twofold down-regulated expression were used for pathway enrichment. The results indicated that the NF-ΚB pathway is closely correlated with PLOD3 (Fig. 7A-B). From these, we hypothesized that the NF-κB pathway was involved in promoting CRC progression by PLOD3. To test this notion, we knocked down PLOD3 in HT29 and SW480 cells and found that its downstream TNF-a, IL6 and IL1β expression levels decreased significantly with the knockdown of PLOD3 (Fig. 7C). NF-κB in the cytoplasm is rapidly phosphorylated upon TNF-α stimulation, resulting in its nuclear translocation and subsequent transcription of related genes. As expected, we observed that the knockdown of PLOD3 dramatically attenuated the nuclear translocation of NF-κB. We analyzed the effect of PLOD3 on NF-κB nuclear translocation using confocal microscopy. As shown in Fig. 7D, the knockdown of PLOD3 in MC38 and SW480 cells led to significant inhibition of NF-κB nuclear translocation. There are 164 Interactors of PLOD3 by BioGRID Version 4.4.225 (https://thebiogrid.org/) (Additional file 1: Figure S1). Among them, IKBKE has been shown to interact with PLOD3 by the Affinity Capture-MS [29]. Subsequently, we performed a luciferase reporter assay, and the results in Fig. 7E indicated that the knockdown of PLOD3 suppressed the p-NF-κB luciferase activity in HT29 cells. We also detected significantly lower levels of phosphorylated NF-κB in PLOD3-knockdown cell lines (Fig. 7F). These data indicate that PLOD3 is important in the phosphorylation and nuclear translocation of NF-κB.

## Discussion

PLODs are procollagen-lysine, 2-oxoglutarate 5-dioxygenases (PLODs) that regulate lysine hydroxylation and collagen stabilization. The PLOD family includes the PLOD1, PLOD2, and PLOD3 genes, which encode LH1, LH2, and LH3 proteins, respectively [30]. These proteins facilitate collagen maturation and secretion by catalyzing the post-translational hydroxylation of lysine residues in pro-collagen molecules. Their functions in the regulation of collagen biosynthesis are related to metastasis. There is growing evidence that most collagen proteins are upregulated in cancer at genetic and protein levels. They all regulate vital steps in tumorigenesis, such as proliferation, apoptosis, angiogenesis, invasion, and metastasis. Therefore, PLOD3 may participate in colon cancer and liver metastasis.

Recent studies have shown that PLOD3 is a potential biomarker for the diagnosis and prognosis of CRC [31]. In addition, PLOD3 might be associated with the “immune desert” phenotype and promote TVA tumorigenesis and colorectal cancer progression [32]. Moreover, Guo et al. reported that COLGALT2 played a pro-carcinogenic role in OvCa and interacted with PLOD3 to promote tumor aggressiveness [33]. These studies suggested that PLOD3 was associated with tumor progression and that upregulation of PLOD3 was also strongly associated with poor prognosis. In addition, PLOD2 activated integrin β1 through IL-6/STAT3 signaling to promote invasion and metastasis in oral squamous cell carcinoma [34]. Although PLOD3 has been evaluated in a variety of cancers, its role in CRC cancers is unclear.

The morbidity and mortality of colorectal cancer patients predominantly result from primary tumor cell invasion and metastasis to secondary sites [35]. We identified the potential role of PLOD3 in colorectal cancer through preliminary bioinformatics screening. In this context, we demonstrated that PLOD3 downregulation interfered with tumor progression and was significantly associated with advanced colorectal cancer. Here, we used public databases such as TCGA and GEO to compare the different expressions of PLOD3 and its survival differences in colon cancer to clarify the effect of PLOD3 in the occurrence and development of colon cancer. We confirmed by immunohistochemical analysis that PLOD3 was significantly upregulated in patients with colon cancer. Clinical data also showed that PLOD3 expression levels significantly correlated with the pathological grade of colorectal cancer. In addition, PLOD3 downregulation improved overall survival in colorectal cancer patients [31], and PLOD3 could be used as a prognostic marker in these patients. We also constructed stable transgenic cells with knockdown and overexpression of PLOD3 for functional studies. The effect of PLOD3 on cell proliferation was investigated by CCK8 and cell counting experiments, and the impact of PLOD3 on cell migration ability was analyzed by transwell assay. Since tumorigenesis in vivo is a very complex process, we also constructed a liver metastasis model to investigate the mechanism of action of PLOD3 in vivo.

Colorectal carcinogenesis is genetically and epigenetically regulated and is associated with the tumor microenvironment (TME), especially the tumor immune microenvironment [1]. In the recent decade, Immune Checkpoint Blockade has attracted much discussion due to its good efficacy in treating solid malignancies such as melanoma and non-small cell lung cancer [36]. Immune Checkpoint Blockade therapies are also used for CRC patients with defective DNA mismatch repair (dMMR)/MSI-H. Investigating the link between CRC and immune cells in the tumor microenvironment may provide a more effective therapeutic strategy for the immunotherapy of CRC. T cells infiltrating the TME are heterogeneous, containing both effector T cells and cytotoxic T cell populations. We observed that when we knocked down PLOD3, the infiltration of cytotoxic T cells in the tumor microenvironment was significantly increased. We also used flow cytometry to detect T cells in the tumor tissue of mice. Then RNA seq was used to find molecules downstream of PLOD3. The first discovery is that PLOD3 interferes with colorectal cancer tumor progression by affecting the nuclear translocation of NF-κB. Our study indicated that knockdown of PLOD3 inhibits NF-κB activation, suggesting that inhibition of NF-κB signaling by PLOD3 may be a promising treatment for colorectal cancer. In evaluating the contributions of our study, several limitations should be acknowledged. Other immune cells were not formally assessed in the TME. Thus far, the mechanism of tumor cell migration and invasion involving PLOD3 is unknown.

In conclusion, our research characterized PLOD3 as a new precancerous metastatic factor for colorectal cancer, a novel oncogene with an important role in the induction of metastasis, and possibly had broader application in colorectal cancer.

### Supplementary Information

**Additional file 1: Table S1.** Correlation between the clinicopathological features and expression of PLOD3. **Figure S1**. There are 164 Interactors of PLOD3 by BioGRID and IKBKE has been shown to interact with PLOD3 by the Affinity Capture-MS.

## Data Availability

The data that support the findings of this study are available from the corresponding author upon reasonable request.

## Abbreviations

CRC: Colorectal cancer
IHC: Immunohistochemistry
GEO: Gene Expression Omnibus
TME: Tumor microenvironment
ssGSEA: Single-sample gene set enrichment analysis
MCP-1: Monocyte chemoattractant protein-1
MMP: Matrix metalloproteinases
PLODs: Procollagen-lysine 2-oxoglutarate 5-dioxygenases
VEGF: Vascular endothelial growth factor
VCAM: Vascular cell adhesion molecules
TCGA: The Cancer Genome Atlas
