# Supplementary material for: PLOD3 facilitated T cell activation in the colorectal tumor microenvironment and liver metastasis by the TNF-α/ NF-κB pathway
Source: J Transl Med. 2024 Jan 6;22:30. doi: 10.1186/s12967-023-04809-w (PMC10771005; doi:10.1186/s12967-023-04809-w)
Supplement: Supplementary file 1 — Additional file 1: Table S1. Correlation between the clinicopathological features and expression of PLOD3. Figure S1. There are 164 Interactors of PLOD3 by BioGRID and IKBKE has been shown to interact with PLOD3 by the Affinity Capture-MS. [file 12967_2023_4809_MOESM1_ESM.docx]

**PLOD3 facilitated T cell activation in the colorectal tumor microenvironment and liver metastasis by the TNF-α/NF-κB pathway**

**Supporting Materials and Methods**

**Library preparation and RNA sequencing (RNAseq)**

This study was approved by the Clinical Institutional Review Board (IRB) of our institutes. We collected CRC patients samples who underwent complete resection. The sample collection was agreed with the guardian of each patient. After the isolation of total RNA and QC procedures, mRNA from eukaryotic organisms is enriched using oligo(dT) beads. For prokaryotic organisms or eukaryotic organisms' long-non-coding libraries, rRNA is removed using the Ribo-Zero kit that cleaves the mRNA. The qualified libraries are fed into Illumina sequencers after pooling according to their effective concentration and expected data volume. A qualified enough amount of total RNA was used for library preparation and RNA sequencing. RNAseq with Illumina sequencers was done by Novogene Co., Ltd. RNAseq was transferred from the sequencing instrument to a bioinformatics server for professional bioinformatics analysis. The statistical analysis software, Partek® Genomics Suite® and IBM SPSS Statistics were used for further statistical analysis.

**Bioinformatic analyses**

Cancer tissues and adjacent tissues of 41 pairs of COAD patients were obtained from the TCGA database, and the microarray datasets (GSE41258, GSE41568 and GSE68468) were from the GEO database (http://www.ncbi.nlm.nih.gov/geo/). And the survival analysis originated from RNA-seq data and clinical data of patients, which were obtained from the TCGA cohort. After screening, 332 COAD cases with survival times and clinical information were chosen. PLOD3 gene expression in selected topics was obtained and then divided into the high PLOD3 expression group (n = 104) and the low PLOD3 expression group (n = 228) according to the optimal cut-off value (using R package survminer). Kaplan–Meier curve was used to depict the survival curve of the two groups, and a log-rank test was performed to analyze the statistical difference between the two groups with the *P*-value calculated.

**Cell migration and invasion assay**

Transwell migration and invasion assay were performed using CRC cells (3×10^4^ cells). For the migration assay, cells were inoculated into the upper chamber (pore size, 8-μm) without matrigel. For the invasion assay, cells were inoculated into the upper chamber with matrigel (BD Biosciences, USA). After 48 hours, any excess cells were removed by gently scraping with a cotton swab. The remaining cells were fixed with 4% paraformaldehyde for 30 minutes and then stained with 0.5% crystal violet for 30 minutes. We randomly selected five fields of view to photograph and count the invading cells using Image J software.

**Clone formation assay**

To perform the clone formation assay, cells in the logarithmic growth phase were digested and plated in a 6-well plate, with 1000 cells per well. After incubating for 48 hours, we observed the cell morphology and counted the number of clones. Culture was continued for 14 days or until clones were visible to the naked eye. The upper medium was then discarded, and the wells were washed twice with PBS. The cells were fixed with 4% paraformaldehyde for 1 hour and stained with crystal violet. We captured photographs and counted the number of clones for statistical analysis.

**Clinical specimens and cell culture**

31 pairs of CRC liver metastasis samples and corresponding adjacent tissues samples and 64 CRC paraffin sections for immunohistochemistry (IHC) and Western blot were obtained with informed consent from Affiliated Drum Tower Hospital, Medical School of Nanjing University (Nanjing, China) and the First Affiliated Hospital of Xi'an Jiaotong University, respectively. The human CRC cell lines (SW620, DLD-1, SW480, HCT116, HT29) and the mouse CRC cell lines (CT26, MC38) were obtained from the American Type Culture Collection. All cell lines in this study tested negative for Mycoplasma. All CRC cells were cultured in DMEM(Gibco,USA), added with 10% FBS and 1% penicillin/streptomycin in the incubator at 37 °C with 5% CO_2_.

**Immunohistochemistry (IHC)**

All the tissues were paraffin-embedded and cut into 6 μm slices. The slides were placed in an oven at 55 ℃ overnight, deparaffinized with xylene, and hydrated in ethanol with different concentrations. Sections were treated with sodium citrate buffer (0.01 mol/L, pH 6.0) to recover antigen and microwaved for 30 min, then 3% H_2_O_2_ was used to block the endogenous catalase. After blocking nonspecific antigen by incubating with 5% BSA, the primary antibody was incubated overnight. Next, sections were incubated in secondary antibody for 2 hours at room temperature, developed with a 3,3-diaminobenzidine (DAB) kit, dehydrated and sealed after counterstaining nuclei with hematoxylin. Finally, slides were observed under a bright-field microscope and data was acquired using an imaging system.

**RNA extraction and quantitative RT-PCR**

Total RNA was extracted from cells and tissues using TRIZOL according to the manufacturer's instructions. RNA was subsequently reverse transcribed to CDNA by All-In-One RT Master Mix (ABM good) and used for subsequent QPCR analysis. The primer sequences were as follows:

GAPDH: forward: 5’-ACCCAGAAGACTGTGGATGG-3’

reverse: 5’-TTCAGCTCAGGGATGACCTT-3’;

PLOD3: forward: 5’- CAACTACACTGTGCGGACCC-3’

reverse: 5’-CTGCCACTCTGGACGAACTT -3’

**Western blot**

PLOD3, **NF-κB** , VIM, CDH1 protein expression levels were analyzed using standard western blotting. Anti- NF-κB (1:1000 dilution ,CST), anti-p- NF-κB (1:1000 dilution) (CST), anti-VIM (1:1000,Abclonal) , anti-CDH1 (1:1000,Abclonal) and anti-GAPDH (1:100000,CST) antibodies was used to measure levels of PLOD3, NF-κB , VIM, CDH1 and GAPDH.

**Flow cytometry**

After euthanizing the mice, tumor tissue and peripheral blood were isolated, and lymphocytes were subsequently isolated with percoll. For flow cytometry staining, first incubate with mouse Fc receptor blocker (anti-mouse CD16/32) for 10 min at 4°C, followed by appropriate surface-labeling antibodies (anti-mouse CD3-FITC, anti-mouse CD3-FITC, Mouse CD69-APC, Anti-mouseCD25-PE, Anti-mouseCD127-BV421) were incubated at 4°C for 30 minutes in the dark. Different cell populations were analyzed using flow cytometry (BD Biosciences) and FlowJo software (BD Biosciences).

**Table S1 Correlation between the clinicopathological features**

**and expression of PLOD3**

|  | **Low expression** | **High expression**  **(N=104)** | **P-value** |
| --- | --- | --- | --- |
|  | **(N=228)** |  |  |
| **Age** |  |  |  |
| Mean (SD) | 66.6 (13.3) | 67.6 (11.8) | 0.523 |
| Median [Min, Max] | 69.0 [32.0, 90.0] | 69.0 [41.0, 89.0] |  |
| Missing | 1 (0.4%) | 1 (1.0%) |  |
| **Gender** |  |  |  |
| female | 105 (46.1%) | 51 (49.0%) | 0.699 |
| male | 123 (53.9%) | 53 (51.0%) |  |
| **Status** |  |  |  |
| Alive | 187 (82.0%) | 71 (68.3%) | **0.00806** |
| Dead | 41 (18.0%) | 33 (31.7%) |  |
| **pT_stage** |  |  |  |
| T1+T2 | 40 (17.5%) | 22 (21.2%) | 0.594 |
| T3+T4 | 187 (82.0%) | 82 (78.8%) |  |
| Tis | 1 (0.4%) | 0 (0%) |  |
| **pN_stage** |  |  |  |
| N0 | 138 (60.5%) | 57 (54.8%) | 0.617 |
| N1 | 54 (23.7%) | 28 (26.9%) |  |
| N2 | 36 (15.8%) | 19 (18.3%) |  |
| **pM_stag** |  |  |  |
| M0 | 166 (72.8%) | 71 (68.3%) | **0.0258** |
| M1 | 24 (10.5%) | 21 (20.2%) |  |
| MX | 34 (14.9%) | 9 (8.7%) |  |
| Missing | 4 (1.8%) | 3 (2.9%) |  |
| **Stage** |  |  |  |
| I | 35 (15.4%) | 18 (17.3%) | 0.0507 |
| II | 95 (41.7%) | 32 (30.8%) |  |
| III | 69 (30.3%) | 27 (26.0%) |  |
| IV | 24 (10.5%) | 21 (20.2%) |  |
| Missing | 5 (2.2%) | 6 (5.8%) |  |

Figure S1

Figure S1 There are 164 Interactors of PLOD3 by BioGRID and IKBKE has been shown to interact with PLOD3 by the Affinity Capture-MS.
